# Remedies and Their Uses

**Published:** 1906-12-08

**Authors:** 


### Remedies and their Uses.

### Kephaldol.

This new febrifuge and anti-neuralgic has recently been announced by Dr. Franz Stohr, Wien-Baden, from whom it may be obtained. It is a complex substance formed by the action of citric and salicylic acid upon phenetidine and combined with quinine; its use was suggested by Ortner, in whose clinic its effects have been observed (Fritsch).[1] The single dose is given as 3.0 to 5.0 gm., and for repeated doses 0.2 to 1.0 gm. in capsules (Stohr). It is a mild but prompt anti-pyretic which gives rise to no unpleasant symptoms and has no ill-effects on the heart; the rapid pulse of fever is slowed to normal, the tone of the arteries improved, and the blood-pressure, both systolic and diastolic, is brought within normal limits. For headache, migraine, and neuralgia of all sorts it has an excellent sedative action, and it possesses an unexpected property among the group of drugs to which it belongs in that it has a marked an-hidrotic effect, and may be advantageously employed in 1-gm. doses in the night-sweats of phthisis. It is also suggested that, combined with salicylate of soda, it may render that drug more easily tolerated in certain cases. The dosage is given in grammes, as Kephaldol is not yet on the English market.

[1] Fritsch, Wiener klin. Wochenschr., xix., 33, p. 1012.

### Taeniol.

A new anthelmintic which is claimed to be not only efficacious for all other intestinal worms, but to be an absolutely certain remedy for ankylostomiasis. The preparation was introduced by Goldmann[1] at the International Conference on the Cure of Ankylostomiasis at Cologne in 1903, and is obtained from the rind of Musenna Abyssinica, a myrsinacea found in Persia. The active principle is not known, but the extract is usually given in combination with thymol. Taeniol is said to act promptly without any undesirable symptoms, save occasionally a slight giddiness, which passes off when the drug is discontinued. After three years' experience Goldmann has recently published further reports which amply justify his first favourable impressions. The method of administration (adults) is as follows: A light diet only should be taken for one day, followed by thorough purging with calomel; and next morning, after a breakfast consisting only of a cup of tea, 13 to 15 capsules (Merck, Darmstadt), should be taken in a little water at intervals of ten minutes, with an interval of an hour after the sixth of eighth capsule; an hour or two after the last capsule further purging with calomel should follow. Owing to taeniol possessing practically no toxic properties, there is no danger to be anticipated from a prolonged stay in the intestine, thus giving the drug an advantage over other anthelmintics that the intestinal parasites are the more thoroughly exposed to its influence before the purging is commenced.

[1] Goldmann, Report of Congress, 1903; Heilmittel revue, 1904, 9-11; Wiener, klin. Wochenschr., 1906, 36, p. 1090; Liamberger, Berl. klin. Wochenschr., 1905, 14, p. 390; Merck's Year-book, 1905.

